# Genomic Comparison between *Salmonella* Gallinarum and Pullorum: Differential Pseudogene Formation under Common Host Restriction

**DOI:** 10.1371/journal.pone.0059427

**Published:** 2013-03-15

**Authors:** Ye Feng, Randal N. Johnston, Gui-Rong Liu, Shu-Lin Liu

**Affiliations:** 1 Genomics Research Center (one of The State-Province Key Laboratories of Biomedicine-Pharmaceutics of China), Harbin Medical University, Harbin, China; 2 Department of Biochemistry and Molecular Biology, University of Calgary, Calgary, Canada; 3 Department of Microbiology and Infectious Diseases, University of Calgary, Calgary, Canada; Indian Institute of Science, India

## Abstract

**Background:**

*Salmonella* serovars Enteritidis and Gallinarum are closely related, but their host ranges are very different: the former is host-promiscuous and the latter can infect poultry only. Comparison of their genomic sequences reveals that Gallinarum has undergone much more extensive degradation than Enteritidis. This phenomenon has also been observed in other host restricted *Salmonella* serovars, such as Typhi and Paratyphi A. The serovar Gallinarum can be further split into two biovars: Gallinarum and Pullorum, which take poultry as their common host but cause distinct diseases, with the former eliciting typhoid and the latter being a dysentery agent. Genomic comparison of the two pathogens, with a focus on pseudogenes, would provide insights into the evolutionary processes that might have facilitated the formation of host-restricted *Salmonella* pathogens.

**Methodologies/Principal Findings:**

We sequenced the complete genome of Pullorum strains and made comparison with Gallinarum and other *Salmonella* lineages. The gene contents of Gallinarum and Pullorum were highly similar, but their pseudogene compositions differed considerably. About one fourth of pseudogenes had the same inactivation mutations in Gallinarum and Pullorum but these genes remained intact in Enteritidis, suggesting that the ancestral Gallinarum may have already been restricted to poultry. On the other hand, the remaining pseudogenes were either in the same genes but with different inactivation sites or unique to Gallinarum or Pullorum, reflecting unnecessary functions in infecting poultry.

**Conclusions:**

Our results support the hypothesis that the divergence between Gallinarum and Pullorum was initiated and facilitated by host restriction. Formation of pseudogenes instead of gene deletion is the major form of genomic degradation. Given the short divergence history of Gallinarum and Pullorum, the effect of host restriction on genomic degradation is huge and rapid, and such effect seems to be continuing to work. The pseudogenes may reflect the unnecessary functions for *Salmonella* within the poultry host.

## Introduction

*Salmonella* are important pathogenic bacteria infecting humans or animals. Different *Salmonella* serovars may either infect a variety of animals or be restricted to a single host. Take serovars from serogroup D for example, Enteritidis can infect multiple hosts whereas Gallinarum can only infect chickens, though they are very similar to each other genetically. Two contrary scenarios in terms of comparative genomics may explain their differences in host range. One is that the Last Common Ancestor (LCA) of Enteritidis and Gallinarum infected chickens only; during evolution, Enteritidis acquired certain mobile genetic elements and thereby became capable of infecting other hosts. The other is that their LCA was a host-generalist; after divergence, Gallinarum evolved to infect birds only, whereas Enteritidis remained the same. Recently, comparative genome analyses have revealed that Gallinarum contained a much greater number of pseudogenes than Enteritidis [1], providing support to the latter scenario.

The serovar Gallinarum can be further divided into two biovars: bv. Gallinarum (abbr. bvGa) and bv. Pullorum (abbr. bvPu). The two biovars are both non-motile and have nearly identical biochemical characteristics except that bvPu can produce rapid decarboxylation of ornithine. Clinically, they both infect chickens only, with bvGa causing typhoid but bvPu causing pullorum disease. We have genotyped a large number of bvGa and bvPu strains by pulsed field gel electrophoresis and found that the cleavage patterns by a series of endonucleases, such as XbaI, AvrII, and SpeI, can be clearly distinguished between the two biovars [2], [3], suggesting that they have separated into distinct lineages in terms of evolution. Given that they are both restricted to infect chicken and that host restriction features genomic degradation, one of the unresolved questions is whether bvPu has undergone the same extensive degradation as bvGa. If so,will the same set of genes be inactivated since their function become superfluous under the same biological niche? In order to address these questions, we sequenced the complete genome of bvPu str. CDC1983-67 and compared it with that of bvPu str. RKS5078, which we sequenced previously but not analyzed [4], bvGa (represented by str. 287/91), Enteriditis (represented by str. P125109) and other *Salmonella* strains.

## Materials and Methods

### Genome Sequencing and Annotation

The strain CDC1983-67 can be obtained from the *Salmonella* Genetic Stock Center (http://www.ucalgary.ca/~kesander). Its genomic sequence was determined by using a shotgun method. We cloned sonicated and size-fractionated DNA into plasmid vectors (2∼4 kbp inserts). Random clones were sequenced by using ABI3730 automated sequencers. The Phred/Phrap/Consed package was used for sequence assembly, and the final coverage reached 4×. The genome of Pullorum str. RKS5078 was used as reference for finishing. Gaps were filled by PCR amplification and primer walking methods. Illumina Solexa sequencing was implemented for quality improvement (80× coverage).

The genomes of CDC1983-67 and other strains used for comparison were submitted to the web service RAST for automatic annotation [5] followed by manual checking. In order to identify pseudogenes, all query genomes were firstly submitted to NCBI online service Microbial Genome Submission Check (http://www.ncbi.nlm.nih.gov/genomes/frameshifts/frameshifts.cgi) for identifying potential pseudogenes caused by frameshift. Then Enteritidis P125109 was used as a reference genome and both bvGa and bvPu genomes were compared with it for finding more pseudogenes within the two biovars. Orthologous genes that contain frameshifts, nonsense mutations, truncations, or indels that altered >20% of the amino acid sequence in comparison with the reference sequence were treated as pseudogenes.

### Genome Comparison

Orthologous relationship of the protein-coding sequences between genomes was determined by using NCBI Basic Local Alignment Search Tool (BLAST), with the criteria being identity >80% and e-value <1e−10. Orthologs commonly present in all genomes were used for constructing genome-scale phylogenetic tree. Briefly, individual orthologs were aligned by using MAFFT [6], back translated to DNA sequences by using bioperl aa_to_dna module, and concatenated to obtain a “chromosomal” alignment. The phylogenetic tree was constructed with the program PHYML under GTR+gamma+I model [7].

The synonymous and non-synonymous substitution rates were calculated with the Nei and Gojobori methods as implemented in codeml program of PAML package v4.1 [8]. Because each gene has few substitutions, the computation of dN and dS is subject to significant imprecision (often both dN and dS are null). Therefore the above concatenated alignment was put into PAML for calculation.

The number of large indels between genomes was presumed to be identical to the number of large conserved blocks, which was estimated by using MUMmer package [9]. Indel rate was defined herein as the number of indels divided by the length of genomic sequences, so it represented the number of indel events per base. The circular map that illustrates the general genomic feature of bvPu str. CDC1983-67 as well as its comparison with other strains were plotted by using the software BLASTring [10].

### Accession Numbers

The genome of Pullorum str. CDC1983-67 was deposited in NCBI database under the accession number CP003786. The Solexa reads from resequencing have been submitted to NCBI Sequence Read Archive under accession number SRX216122.

## Results and Discussion

### General Comparison between *S.* Pullorum and other *Salmonella*

**Figure 1:**
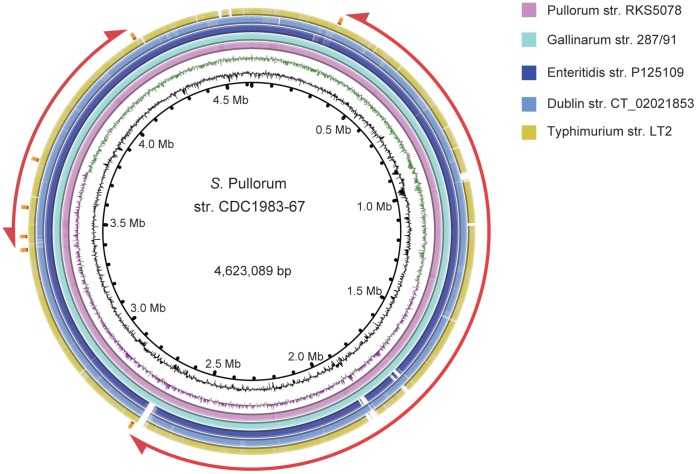
Circular Map of Pullorum str. CDC1983-67 genome. Circles range from 1 (inner circle) to 8 (outer circle): 1, coordinates of the genome; 2, GC content; 3, GC skew; 4–8, comparison of gene content with bvPu strain RKS5078, bvGA, Enteritidis, Dublin and Typhimurium, respectively. The locations of seven rRNA operons are indicated by the small outer orange blocks. The outmost arcs represent the chromosomal rearrangements between bvGa and bvPu (the larger one) and between bvGa and Enteritidis (the smaller one).

The genome of str. CDC1983-67 is 4,623,089 bp long. This size is smaller than all host-generalist *Salmonella* genomes previously reported and is similar to those of bvGA and the host-strict serovar Paratyphi A. The average GC content is 52.2% and the replication origin and terminus, predicted by GC-skew, are near 1.373 and 3.722 Mb, respectively. It contains seven ribosomal RNA operons, 75 transfer RNAs, and 4,443 protein-coding genes with average length being 867 bp. All these features are very similar to those of other *Salmonella* genomes, and a predominant similarity and synteny of genomes was present between serogroup D strains, despite several rearrangements mediated by rRNA operons (Figure 1).

**Figure 2:**
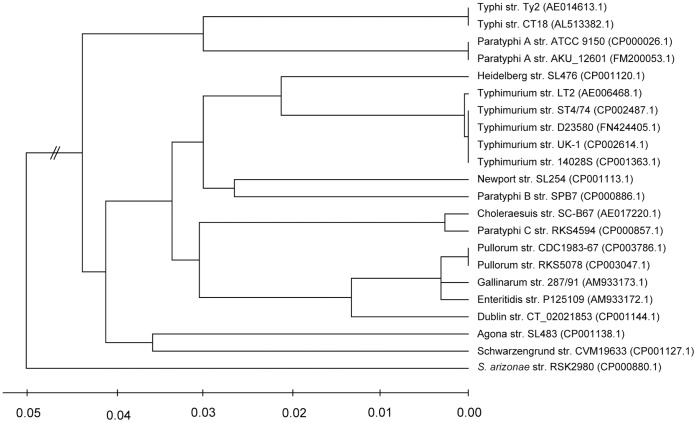
Maximum Likelihood Tree for *Salmonella enterica* strains. Genes that are conserved in all strains were aligned and concatenated for tree construction. In the brackets are the accession numbers of these genomes downloaded from NCBI database. A scale bar for the genetic distance is shown at the bottom.

For a deeper comparative analysis, we concatenated all conserved genes and constructed the phylogenetic tree (Figure 2). As expected, all Gallinarum strains were clustered together and were next close to Enteritidis and Dublin in order. Inside the Gallinarum/Pullorum complex, the two bvPu strains had nearly identical nucleotide sequences, having diverged from bvGa quite recently. Coincidentally, the genetic distance between Paratyphi C (a human-adapted serovar) and Choleraesuis (a swine-adapted serovar) was similar to that between Enterididis and Gallinarum. Together with the fact that the Typhi clone emerged at the hunter-gatherer phase of human history [11], [12], it is tempting to question why all of these host-adapted serovars emerged recently. It is likely that human speciation and the domestication of animals helped shape the host-specificities of *Salmonella*: when *Salmonella* invaded the human community, some clones evolved to become adapted to the human host, whereas some others infected the domesticated animals and became closely associated with them. However, this speculation needs to be validated by further evidence.

**Figure 3:**
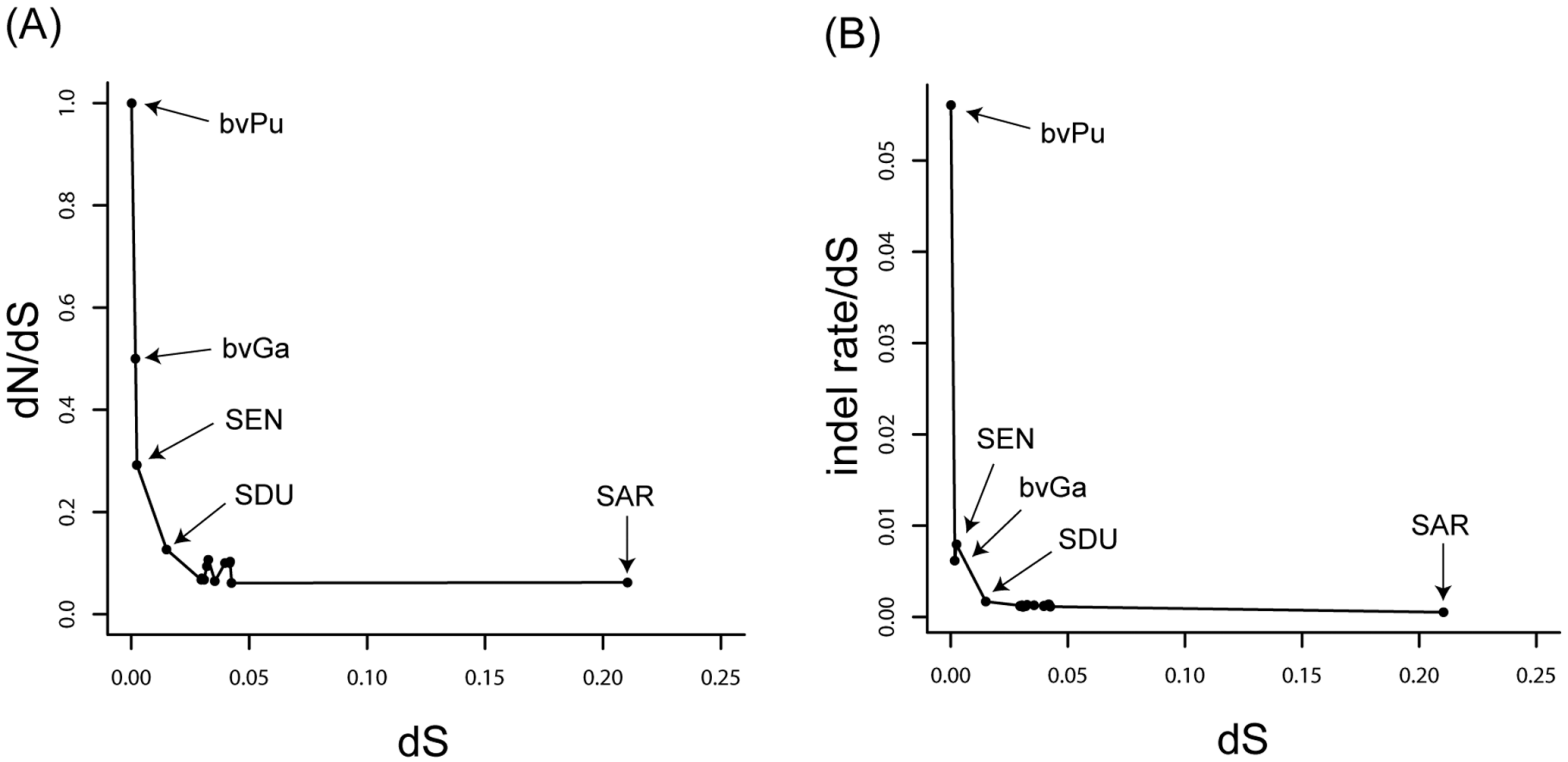
Relationship between bvPu str. CDC1983-67 and other *Salmonella* strains. (A) Association between dN/dS (y axis) and dS (x axis). dS represents synonymous substitution rate and dN represents non-synonymous substitution rate. (B) Association between indel/dS (y axis) and dS (x axis). The points in the plot are: bvPu, bvPu str. RKS5078; bvGa, bvGa str. 287/91; SEN, Enteritidis str. P125109; SDU, Dublin str. CT_02021853; SAR, *S. arizonae* str. RSK2980; other points represent the *S. enterica* subspecies I strains used for comparison in this study.

We also measured rates of synonymous substitution, non-synonymous substitution and indels (short for insertion and/or deletion) by comparing str. CDC1983-67 with other strains. The synonymous substitution rate was used to measure the genetic distance since it is little affected by the selection that comes from functional restraint. With the increase of the genetic divergence, both the non-synonymous substitution rate and the indel rate would drop quickly first and then reach equilibrium (Figure 3). It has been recognized that dN/dS ratio might decrease over time due to a lag in the removal of slightly deleterious non-synonymous mutations [13]. Also, the rate of horizontal transfer was very high in the early stages of prokaryotic evolution, and yet most of the genes gained were deleted again rapidly due to their nature of being deleterious [14]. In our analysis, the turning point of the curves corresponds approximately to the delimiting line of serogroup, so serogroup is very likely to be a taxonomical unit that has practical significance during evolution of *Salmonella*.

The difference of gene content between serogroup D strains was small. A total of 125 genes were present in serovar Enteritidis but absent from serovar Gallinarum (see Table S1). Most of them were phage-related genes, including those encoding fimbriae and type III secreted proteins. Meanwhile, most of the 125 genes could also be found in other *Salmonella* serovars, especially in Dublin, whereas only 23 genes were really unique to Enteritidis (Table S1), indicating that it was Gallinarum that lost certain genes during host restriction rather than that Enteridis acquired some genes and became host-generalist. There were minor differences between bvGa and bvPu: 14 genes were present in bvGa but absent from bvPu, while three genes were present in bvPu but absent from bvGa (Table S1). The roles of these genes are still unclear, but they are probably not sufficient to explain the phenotypic difference between the two biovars.

### Comparison of Pseudogenes among Serogroup D Strains

We focused our analysis on pseudogenes since pseudogene formation has been considered as hallmark of host-restricted *Salmonella* [1], [15], [16], [17], [18], [19], [20]. Enteritidis, bvGa, bvPu RKS5078 and bvPu CDC1983-67 possessed 48, 215, 263 and 240 pseudogenes, respectively. Comparison between bvGa and bvPu revealed that the two biovars shared 75 pseudogenes, 60 of which had the same inactivation sites and the rest 15 pseudogenes had different inactivation sites (see Table S2). The former pseudogenes had probably been formed before the divergence of the two biovars and the same inactivation sites could as a result be inherited to the offspring; in contrast the latter pseudogenes might have lost functions after the divergence. Because both bvGa and bvPu have chicken as their unique host, it is very likely that their last common ancestor had already been restricted to chickens. So it is reasonable to believe that these pseudogenes all reflect the superfluous functions for infecting chickens. Meanwhile, the two bvPu strains shared 113 biovar-specific pseudogenes which had the same inactivation sites in the two bvPu strains. Given the close genetic relationship between bvGa and bvPu, accumulation of a large number of pseudogenes within such a short divergence time suggests that the effect of host restriction on pseudogene formation was quite strong.

Of the pseudogenes, *flhB* and *flgK* could explain why serovar Gallinarum lacked flagella. The gene *emrB* encodes a multidrug resistance protein. Disruption of this gene does not necessarily mean that bvPu live without selective pressure from antibiotics. Other genes may have better abilities to resist antibiotics so that *emrB* does not have to play its role. Notably, many outer membrane protein-encoding genes appear in the list of pseudogenes. Previous literature has predicted the association between OmpC and host-specificity on the basis of bioinformatic analysis [21]. Although detailed roles of the outer membrane proteins in bacterial infection are largely unknown, lack of them may result in the reduction of host range. In addition, many genes involved in the synthesis and transport of sugar and protein were also degraded. This phenomenon is quite like the situation in organisms such as *Buchnera aphidicola* and *Mycoplasma pneumoniae* [22], [23]. The obligate lifestyle guarantees a stable niche in which most metabolites can be provided by the host cell. Consequently, most of the metabolic pathways have been totally removed from their genomes and these organisms tend to have a highly reduced genome.

Another important pseudogene for bvPu is *mutL*. MutL is a DNA mismatch repair protein, defects of which lead to greatly increased mutation rate of the bacteria [24]. We believe that, after inactivation of *mutL*, pseudogenes would be generated at a greater pace. The host-restricted pathogens usually have a small effective population size and is subject to increased genetic drift and reduced efficacy of selection. As a result, some less deleterious mutations may be irreversibly accumulated within the genome, leading to a progressive loss of fitness. This ongoing process, known as Muller’s ratchet, may result in bacterial extinction. If so, when bvPu is extinct, will the niche of bvPu be occupied by a new obligate organism? We postulate that this situation might happen in the not very distant future. In fact, bvGa and bvPu have almost been eradicated by vaccine in some countries, and Enteritidis has become the top *Salmonella* etiological agent for poultry. One day, certain strains of Enteritidis might evolve to become the new poultry-adapted *Salmonella* in place of bvGa or bvPu or both.

We also compared the pseudogenes from serovar Gallinarum with those from serovar Dublin since the Dublin bacteria, while infecting both humans and cattle as the primary hosts, are not deemed host-generalist due to their very narrow host range. A total of 114 genes were inactivated in Dublin but remained intact in Enteritidis, with 24 of them being also pseudogenes in serovar Gallinarum (see Table S3). Meanwhile, comparison of pseudogenes between serovar Gallinarum and the human-restricted serovars (Typhi and Paratyphi A) revealed that the fimbiral genes *stbC*, *stfF* and *sthE* and the type III secretion system genes *sifB* and *sopA* were inactivated in all these serovars. These results suggest that the habitats of humans, cattle and chicken share certain features that may favor functional loss of the same set of genes. It is worth noting that host-restricted *Salmonella* tend to cause systemic infections in their host rather than gastroenteritis, which is usually caused by host-generalist serovars. So host restriction may necessarily be associated with the conversion from gut to systemic infection lifestyle. As such, the gene degradation events may be parts of the niche adaption process. For example, loss of fimbriae leads to a relatively smoother cell surface and thereby prevents the bacteria from eliciting the host’s pro-inflammatory responses. In the meantime, the bacteria develop the mechanisms to enhance their capability of systemic infection.

## Conclusions

In summary, Enteritidis, bvGa and bvPu are so similar to one another in terms of their main genomic features that they can almost be considered as variants of the same bacteria. Due to host restriction, these bacteria had reduced chances for genetic exchange and began to diverge into different pathogenic lineages. Their minor genomic difference reflects how bacterial genome evolves during the initial stage of the diverging process. Host restriction indeed contributes to functional loss of genes, and formation of pseudogenes instead of gene deletion is the major form. Meanwhile, the large numbers of biovar-specific and strain-specific pseudogenes suggest a huge pool of unnecessary genes for causing systemic infection in chicken. Therefore, more events of gene loss are anticipated to occur in bvGa and bvPu in the future.

## Supporting Information

Table S1 Comparison of gene content among *Salmonella* serogroup D strains.
(XLS)

Table S2 Pseudogenes in *Salmonella* serogroup D strains.
(XLS)

Table S3 Genes that are intact in serovar Enteritidis but are inactivated in serovar Dublin.
(XLS)
